# Vaccination

**Published:** 1881-03

**Authors:** 


VACCINATION.—We publish in another part of the journal, a brief but highly interesting article on the above subject. The method of performing the operation is a practical and common sense one, and the results reached by it in Dr. Dozier's experience, clearly establish its superiority over those generally in vogue. Our own experience is also decidedly in favor of bovine virus, and when it is used successfully we have implicit confidence in its prophylactic influence. But to render protection against small pox almost absolutely certain, it is advisable to renew vaccination, at intervals of several years. Should it be carefully performed a second time, and no result follow, the proof would be nearly positive that the system was perfectly protected from small pox; and as the operation is so slight, there are very few persons who would object to its repetition.

